# Fractional flow reserve or optical coherence tomography for angiographically intermediate coronary stenoses: 5-year outcomes in the FORZA trial

**DOI:** 10.1093/eurheartj/ehae290

**Published:** 2024-06-07

**Authors:** Francesco Burzotta, Andrea Zito, Cristina Aurigemma, Enrico Romagnoli, Francesco Bianchini, Emiliano Bianchini, Lazzaro Paraggio, Carolina Ierardi, Filippo Crea, Antonio Maria Leone, Carlo Trani

**Affiliations:** Department of Cardiovascular Sciences, Fondazione Policlinico Universitario A. Gemelli IRCCS, Largo A. Gemelli 1, 00168 Rome, Italy; Department of Cardiovascular and Thoracic Sciences, Catholic University of the Sacred Heart, Largo A. Gemelli 1, 00168 Rome, Italy; Department of Cardiovascular and Thoracic Sciences, Catholic University of the Sacred Heart, Largo A. Gemelli 1, 00168 Rome, Italy; Department of Cardiovascular Sciences, Fondazione Policlinico Universitario A. Gemelli IRCCS, Largo A. Gemelli 1, 00168 Rome, Italy; Department of Cardiovascular Sciences, Fondazione Policlinico Universitario A. Gemelli IRCCS, Largo A. Gemelli 1, 00168 Rome, Italy; Department of Cardiovascular and Thoracic Sciences, Catholic University of the Sacred Heart, Largo A. Gemelli 1, 00168 Rome, Italy; Department of Cardiovascular and Thoracic Sciences, Catholic University of the Sacred Heart, Largo A. Gemelli 1, 00168 Rome, Italy; Department of Cardiovascular Sciences, Fondazione Policlinico Universitario A. Gemelli IRCCS, Largo A. Gemelli 1, 00168 Rome, Italy; Department of Cardiovascular Sciences, Fondazione Policlinico Universitario A. Gemelli IRCCS, Largo A. Gemelli 1, 00168 Rome, Italy; Department of Cardiovascular and Thoracic Sciences, Catholic University of the Sacred Heart, Largo A. Gemelli 1, 00168 Rome, Italy; Ospedale Isola Tiberina - Gemelli Isola, Via di Ponte Quattro capi 39, 00186 Rome, Italy; Department of Cardiovascular and Thoracic Sciences, Catholic University of the Sacred Heart, Largo A. Gemelli 1, 00168 Rome, Italy; Ospedale Isola Tiberina - Gemelli Isola, Via di Ponte Quattro capi 39, 00186 Rome, Italy; Department of Cardiovascular Sciences, Fondazione Policlinico Universitario A. Gemelli IRCCS, Largo A. Gemelli 1, 00168 Rome, Italy; Department of Cardiovascular and Thoracic Sciences, Catholic University of the Sacred Heart, Largo A. Gemelli 1, 00168 Rome, Italy

**Keywords:** Coronary intermediate lesions, Optical coherence tomography, Fractional flow reserve, Percutaneous coronary intervention

**This paper was guest edited by Anthony N. DeMaria**

## Introduction

Patients with angiographically intermediate coronary lesions (AICLs) have traditionally been managed with coronary angiography, which lacks information on lesion characterization and myocardial ischaemia, thereby hindering an accurate assessment of lesion severity. To fill this gap, adjunctive tools allowing an improved evaluation of lesion severity have been developed.

Fractional flow reserve (FFR) is an invasive index providing physiological information about stenosis and representing the standard of care for guiding the management of AICLs.^1^ Concurrently, intravascular imaging devices, such as optical coherence tomography (OCT) or intravascular ultrasound (IVUS), contribute to percutaneous coronary intervention (PCI) planning and optimization and, recently, have been tested as an alternative to FFR for the management of AICLs.

The FORZA (Fractional Flow Reserve vs. Optical Coherence Tomography to Guide Revascularization of Intermediate Coronary Stenoses) was the first trial to compare FFR and OCT guidance.^2^ In this paper, we report the clinical outcomes observed in the FORZA trial at 5-year follow-up.

## Methods

The FORZA (NCT01824030) trial is an open-label, single-centre, prospective, randomized trial comparing clinical outcomes in patients with at least one AICL randomly assigned in a 1:1 ratio to OCT or FFR guidance for both PCI performance and, in the case of revascularization, optimize PCI results. An AICL was defined as a coronary lesion with a visually estimated percentage diameter stenosis of 30%–80% in the non-distal segment of a major epicardial vessel. The study design with details on the guidance protocols and results at 1-month and 13-month follow-up was previously reported.^2–4^ Follow-up through telephone or clinic visits was systematically performed. Analyses at 5-year follow-up were not pre-specified. The study was approved by the ethics committee of our Institution (code 6261/13) and all patients signed a dedicated informed consent form.

The original primary outcome of the study was a composite of significant residual angina and major adverse cardiac events (MACEs), defined as a composite of all-cause death, myocardial infarction (MI), or target vessel revascularization (TVR) at 13 months.^4^ Since the angina questionnaires were not administered after 13 months, MACEs were selected as the study endpoint in this 5-year outcome analysis.

The Kaplan–Meier method was used to characterize the time until the first event for the primary outcome. Hazard ratios (HRs) and 95% confidence intervals (CIs) were computed with the Cox proportional hazards time-to-event analyses. *Post hoc* subgroup analyses for the primary outcome were performed according to sex, age, diabetes mellitus, multi-vessel disease, acute coronary syndrome (ACS), and left ventricular ejection fraction (LVEF).

## Results

### Baseline characteristics of the population

A total of 350 patients with 446 AICLs in 420 vessels were randomly assigned to OCT (*n* = 174) or FFR (*n* = 176). The baseline clinical and angiographic characteristics of the study population were previously reported and appear to be well matched among groups.^3,4^ The mean age of patients was 69 years, 35.4% had diabetes mellitus, 19.4% presented with ACS, and mean LVEF was 58%. Multi-vessel disease was present in 58.3% of patients and the target vessel was the left anterior descending artery in 81.1% of patients. The rate of patients undergoing PCI was higher in the OCT group than in the FFR group (52.7% vs. 32.4%, *P* < .001). Compared with patients in the FFR group, patients in the OCT group were implanted with more stents (0.64 ± 0.70 vs. 0.33 ± 0.57, *P* < .0001), had larger stent diameter (3.2 ± 0.5 vs. 2.9 ± 0.3, *P* = .009), received more contrast media (280 ± 129 vs. 245 ± 137, *P* = .004), and experienced a higher occurrence of acute kidney injury (8.6% vs. 1.7%, *P* = .034).

### Five-year clinical outcomes

**Figure 1:**
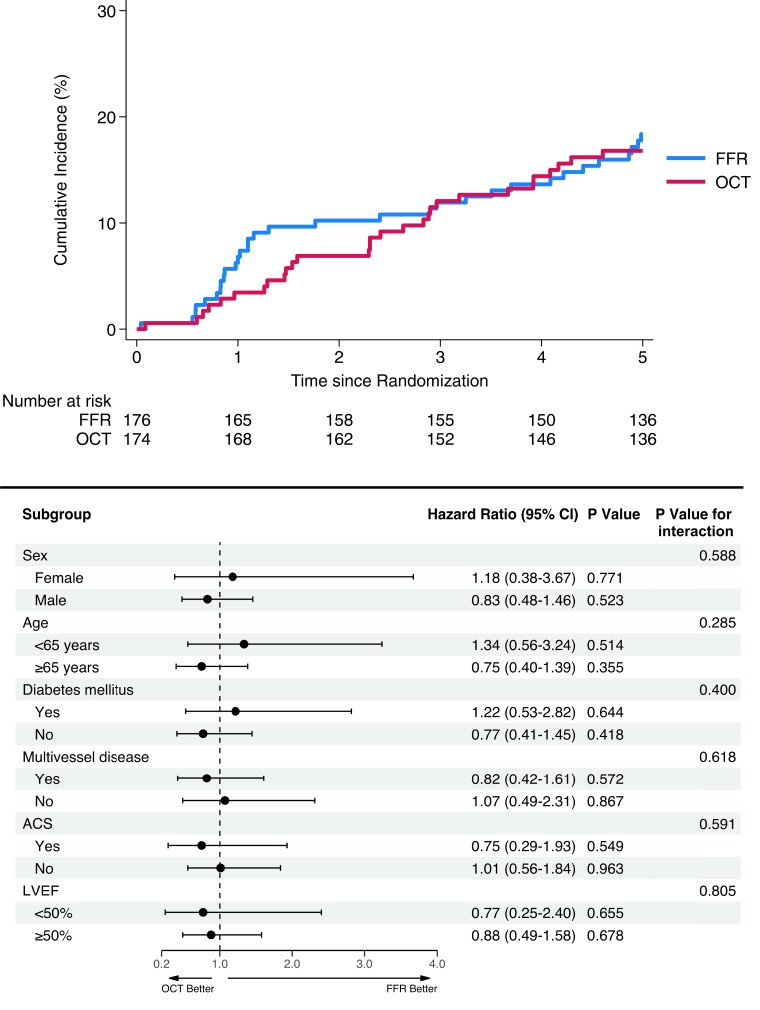
Kaplan–Meier and subgroup analyses for major adverse cardiac events at 5 years. ACS, acute coronary syndrome; CI, confidence interval; FFR, fractional flow reserve; LVEF, left ventricular ejection fraction; OCT, optical coherence tomography

Fifteen patients were lost to clinical follow-up and up to 95.7% of patients completed the 5-year follow-up. At a median follow-up of 1825 (interquartile range 1825–1825) days, MACE occurred in 30 patients in the OCT group and in 33 patients in the FFR group (17.2% vs. 18.8%; HR 0.91, 95% CI 0.55–1.50, *P* = .704) (*Figure 1*).

Compared with the FFR group, the OCT group had numerically lower rates of all-cause death (8.6% vs. 10.8%; HR 0.80, 95% CI 0.41–1.58, *P* = .525), MI (1.1% vs. 2.8%; HR 0.40, 95% CI 0.08–2.07, *P* = .275), and TVR (8.0% vs. 8.5%; HR 0.93, 95% CI 0.45–1.93, *P* = .854).

Results of the primary outcome were largely consistent across subgroups (*Figure 1*).

## Discussion

This study reporting the 5-year results of the FORZA trial showed that, in patients with AICLs, OCT and FFR guidance provide a similar rate of MACE. At 1-month follow-up,^3^ FFR guidance was associated with a higher rate of medical management than OCT guidance; nevertheless, at 13-month follow-up,^4^ the composite outcome of MACE or significant angina was lower with OCT guidance than with FFR guidance. These results support the use of OCT as a valuable alternative to FFR for the management of patients with AICLs and call for additional investigation of the angiographic or clinical settings where OCT and FFR have higher potential.

The introduction of adjunctive tools to angiography has markedly improved the decision-making process regarding revascularization of patients with AICLs and the optimization of PCI results. Indeed, both FFR-guided PCI and OCT-guided PCI have been reported to improve clinical outcomes as compared with angiography-guided PCI.^1,5^ These tools integrate angiography by providing different prognostic indicators such as physiological significance for FFR and plaque burden and lesion characteristics for intravascular imaging devices.^6,7^ In this context, the comparative clinical efficacy of FFR-guided and imaging-guided PCI for patients with AICLs remains a relevant clinical question, actually addressed by two other randomized clinical trials (RCTs) reporting results at a maximum follow-up of 24 months and showing a similar rate of clinical outcomes between IVUS and FFR guidance.^8,9^

The FORZA trial was designed without data linking imaging criteria with clinical outcomes or RCTs evaluating the clinical impact of correcting residual major anatomical defects post-stenting. Nevertheless, the criteria for PCI optimization adopted in the FORZA trial were similar to those of recent RCTs testing an imaging-guided PCI approach.^10^

Overall, the FORZA results should be applied to the target population included, principally composed of patients with chronic coronary syndrome and more than one-half with single-vessel disease. The main limitation of our study is the relatively small sample size with few cardiovascular events, yielding wide CIs. In addition, the primary composite outcome of MACE or significant angina reported at 13-month follow-up was carried over to the clinical outcome of MACE because of unreliable symptom assessment at long-term follow-up.

## Data Availability

Data are available upon reasonable request to the corresponding author (Francesco Burzotta, e-mail: francesco.burzotta@unicatt.it).

## Pre-registered Clinical Trial Number

NCT01824030
